# Risk of subsequent biliary malignancy in patients undergoing cyst excision for congenital choledochal cysts

**DOI:** 10.1111/j.1440-1746.2012.07260.x

**Published:** 2013-01-22

**Authors:** Taku Ohashi, Toshifumi Wakai, Masayuki Kubota, Yasunobu Matsuda, Yuhki Arai, Toshiyuki Ohyama, Kengo Nakaya, Naoki Okuyama, Jun Sakata, Yoshio Shirai, Yoichi Ajioka

**Affiliations:** * Division of Digestive and General Surgery, Niigata University Graduate School of Medical and Dental SciencesNiigata, Japan; † Division of Pediatric Surgery, Niigata University Graduate School of Medical and Dental SciencesNiigata, Japan; § Division of Gastroenterology and Hepatology, Niigata University Graduate School of Medical and Dental SciencesNiigata, Japan; ‡ Division of Molecular and Diagnostic Pathology, Niigata University Graduate School of Medical and Dental SciencesNiigata, Japan

**Keywords:** biliary cancer, congenital choledochal cysts, cyst excision, prognosis

## Abstract

**Background and Aim:**

The aim of this study was to elucidate the risk of subsequent biliary malignancy in patients undergoing cyst excision for congenital choledochal cysts.

**Methods:**

A retrospective analysis of 94 patients who had undergone cyst excision for congenital choledochal cysts was conducted. The median age at the time of cyst excision and median follow-up time after cyst excision were 7 years and 181 months, respectively.

**Results:**

Biliary tract cancer developed in four patients at 13, 15, 23, and 32 years after cyst excision. The cumulative incidences of biliary tract cancer at 15, 20, and 25 years after cyst excision were 1.6%, 3.9%, and 11.3%, respectively. The sites of biliary tract cancer were the intrahepatic (*n* = 2), hilar (*n* = 1), and intrapancreatic (*n* = 1) bile ducts. Of the four patients with biliary tract cancer after cyst excision, three patients underwent surgical resection and one patient received chemo-radiotherapy. The overall cumulative survival rates after treatment in the four patients with biliary tract cancer were 50% at 2 years and 25% at 3 years, with a median survival time of 15 months.

**Conclusions:**

The risk of subsequent biliary malignancy in patients undergoing cyst excision for congenital choledochal cysts seems to be relatively high in the long-term. The risk of biliary malignancy in the remnant bile duct increases more than 15 years after cyst excision. Despite an aggressive treatment approach for this condition, subsequent biliary malignancy following cyst excision for congenital choledochal cysts shows an unfavorable outcome.

## Introduction

A condition that predisposes to extrahepatic cholangiocarcinoma is congenital cystic dilatation or choledochal cyst.^1^–^3^ Although these cysts are relatively rare, coexisting carcinoma as a complication has been well documented.^4^ A mixture of bile and pancreatic secretions may promote the development of carcinoma because 90% of choledochal cysts are associated with anomalous pancreaticobiliary ductal junction (APBDJ).^5^ Carcinoma can develop anywhere along the biliary tract in addition to originating in the choledochal cyst.

Cyst excision is the treatment of choice for congenital choledochal cysts because of the risk of subsequent biliary malignancy.^6^,^7^ A number of authors have reported biliary tract cancer after cyst excision for congenital choledochal cysts;^8^–^30^ however, most previous reports were single cases^8^–^17^ or studies with a limited number of patients.^18^–^30^ The aim of this study was to elucidate the risk of subsequent biliary malignancy in patients undergoing cyst excision for congenital choledochal cysts.

## Methods

### Patients

A total of 114 consecutive Japanese patients with congenital choledochal cysts were identified from a database at Niigata University Medical and Dental Sciences from January 1971 through December 2006. Of the 114 patients, 20 patients who had a coexisting biliary tract cancer including gallbladder cancer (*n* = 19) and extrahepatic cholangiocarcinoma (*n* = 1) were excluded from this study. The remaining 94 patients who underwent cyst excision constituted the final group of patients for this retrospective study. The group comprised 73 females and 21 males with a median age of 7 years (range, 0–63 years) at the time of cyst excision. The protocol of the present study was approved by the Institutional Review Board of Niigata University Medical and Dental Hospital.

Of the 94 patients who underwent cyst excision, 85 patients (90%) had choledochal cysts with APBDJ. According to Todani's classification,^31^ 49 were classified as type I cysts and 45 as type IV-A cysts. Excision of the choledochal cyst was performed as the standard surgical procedure in 48 patients with type I cysts and 45 patients with type IV-A cysts. In the remaining one patient with a type I cyst, a combined partial hepatectomy and cyst excision was performed due to suspicion of coexisting gallbladder carcinoma before surgery. Hepaticojejunostomy, hepaticoduodenostomy, and hepaticoduodenostomy with jejunal interposition were performed as a reconstructive procedure in 86, six, and two patients, respectively.

### Patient follow-up

The median follow-up time after cyst excision for congenital choledochal cysts was 181 months (range, 7–484 months). By the time of disease status assessment, three patients had died of subsequent biliary malignancy following cyst excision. One patient was alive with intrahepatic cholangiocarcinoma following cyst excision, and the remaining 90 patients were alive with no evidence of disease.

### Review of the literature

The English language literature (PubMed, National Library of Medicine, Bethesda, MD, USA), from January 1966 through December 2011, was reviewed using the following Medical Subject Heading (MeSH) terms: “choledochal cyst” or [“cysts/surgery” and “bile duct”] in combination with “cholangiocarcinoma,” “bile duct neoplasms,” “liver neoplasms,” “postoperative complications,” “treatment outcome” and “follow-up study.” The references from the included articles were searched to identify additional cases, and revealed that a total of 30 patients who had undergone choledochal cyst excision (two of whom were included in our earlier reports^11^,^17^) suffered from subsequent biliary malignancy following cyst excision.^8^–^30^

### Statistical analysis

Medical records and survival data were obtained for all the 94 patients. The causes of death were determined based on the medical records. The Kaplan–Meier method was used to estimate both the cumulative incidence of subsequent biliary malignancy following cyst excision and the cumulative patient survival rates after treatment for subsequent biliary malignancy following cyst excision. All statistical analyses were performed using PASW Statistics 17 software (SPSS Japan, Tokyo, Japan). All tests were two-sided, and *P* < 0.05 was considered to be statistically significant.

## Results

### Cumulative incidence of subsequent biliary malignancy following cyst excision for congenital choledochal cysts

**Figure 1:**
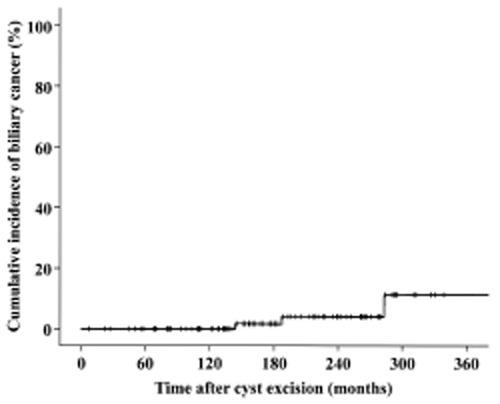
The cumulative incidence of biliary tract cancer after cyst excision for congenital choledochal cysts among 94 patients in the current series. The cumulative incidences of biliary tract cancer were 1.6%, 3.9%, and 11.3% at 15, 20, and 25 years after cyst excision, respectively.

Four of 94 patients (4.3%) had subsequent biliary malignancy following cyst excision for congenital choledochal cysts at 13, 15, 23, and 32 years after surgery during the follow-up period (Table 1). The anatomical sites of biliary tract cancer were the intrahepatic (*n* = 2), hilar (*n* = 1), and intrapancreatic (*n* = 1) bile ducts. The cumulative incidences of biliary tract cancer were 1.6%, 3.9%, and 11.3% at 15, 20, and 25 years after cyst excision, respectively (Fig. 1).

**Table 1:** Characteristics of the four patients with subsequent biliary malignancy following choledochal cyst excision in the current series

Case	Gender	Choledochal cyst		Subsequent biliary malignancy after cyst excision			
		Age at cyst excision (years)	Type of cyst†	Age at detection (years)	Anatomical site	Treatment	Outcome (months)
1	F	14	IV-A	27	Intrapancreatic	Pancreaticoduodenectomy	15; DOD
2	M	12	IV-A	44	Intrahepatic	Left trisectionectomy	35; DOD
3	M	50	I	65	Hilar	Left trisectionectomy	9; DOD
4	F	15	I	38	Intrahepatic	Chemo-radiotherapy	43; AWD

† According to Todani's classification.^31^

AWD, alive with disease; DOD, died of disease.

### Treatment outcome for subsequent biliary malignancy following cyst excision for congenital choledochal cysts

Of the four patients with subsequent biliary malignancy following cyst excision, three patients underwent surgical resection (Table 1). Surgical resection procedures included left trisectionectomy (*n* = 2) and pancreaticoduodenectomy (*n* = 1). Adenocarcinoma was identified as the primary tumor in these three patients. The tumors were well differentiated in Case 1 and moderately differentiated in the others. The tumor of Case 1 showed direct invasion to the duodenum and pancreas without lymph node metastases. The tumor of Case 2 showed invasion to the main portal vein without lymph node metastases. The tumor of Case 3 had periaortic lymph node metastases. These three patients died of disease 9, 15, and 35 months after surgical resection of the subsequent biliary malignancy following cyst excision.

**Figure 2:**
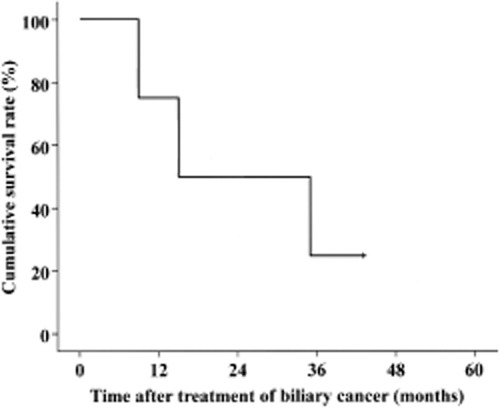
Kaplan–Meier survival estimates in four patients with subsequent biliary malignancy following choledochal cyst excision. The overall cumulative survival rates after treatment were 50% at 2 years post-treatment and 25% at 3 years post-treatment, with a median survival time of 15 months.

The remaining patient (Case 4 in Table 1) underwent exploratory laparotomy. Histological examination of excisional biopsy specimen of an inferior mediastinal lymph node showed moderately differentiated adenocarcinoma. Radical resection was contraindicated due to distant nodal disease. The patient (Case 4) was still alive with intrahepatic cholangiocarcinoma after receiving chemo-radiotherapy. The overall cumulative survival rates after treatment in the four patients with subsequent biliary malignancy following cyst excision were 50% at 2 years post-treatment and 25% at 3 years post-treatment, with a median survival time of 15 months (Fig. 2).

### Review of the literature on subsequent biliary malignancy following choledochal cyst excision

**Figure 3:**
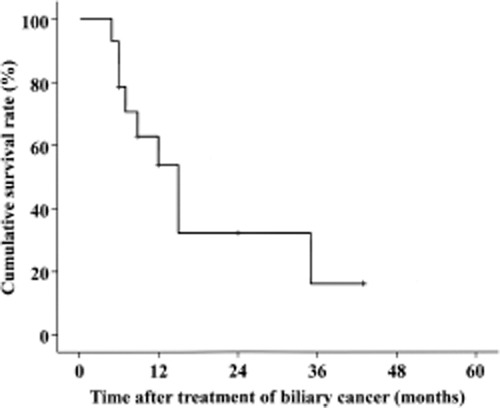
Kaplan–Meier survival estimates in 14 patients identified from the literature review (including our four patients) suffering from subsequent biliary malignancy following choledochal cyst excision with documented outcomes after treatment. The overall cumulative survival rates after treatment were 32% at 2 years post-treatment and 16% at 3 years post-treatment, with a median survival time of 15 months.

An analysis of the 32 reported patients (including our two previously reported cases^11^,^17^ and the two new cases from our present study) with subsequent biliary malignancy following choledochal cyst excision revealed that the anatomical sites of biliary tract cancer were the hilar (*n* = 17), intrahepatic (*n* = 9), and intrapancreatic (*n* = 6) bile ducts (Table 2). Thus, the hilar region was the predominant site of subsequent biliary malignancy following cyst excision. The median interval between cyst excision and the detection of this complication was 6 years (range, 1–34 years). Of the 32 reported cases, 12 patients were treated with supportive care and 14 patients received either surgical resection (*n* = 11), chemo-radiotherapy (*n* = 2) or chemotherapy (*n* = 1) (Table 2). There were no 4-year survivors among the 32 patients with subsequent biliary malignancy following cyst excision. In the 14 patients who received treatment for subsequent biliary malignancy following cyst excision, the overall cumulative survival rates after treatment were 32% at 2 years post-treatment and 16% at 3 years post-treatment, with a median survival time of 15 months (Fig. 3).

**Table 2:** Characteristics of 32 patients with subsequent biliary malignancy following choledochal cyst excision: A literature review

Variable	No. patients
Age at cyst excision (years)†	28 (0.4–68)
Age at detection of biliary malignancy (years)†	41 (18–70)
Gender (M/F/ND)	8/18/6
Type of choledochal cyst (I/IV-A/ND)‡	17/12/3
Site of subsequent biliary malignancy	
Hilar	17
Intrahepatic	9
Intrapancreatic	6
Treatment for subsequent biliary malignancy	
Surgical resection	11
Chemotherapy or chemo-radiotherapy	3
Supportive care	12
ND	6
Survival status	
DOD	24
NED	5
DOO	2
AWD	1

† Values are median (range).

‡ According to Todani's classification.^31^

AWD, alive with disease; DOD, died of disease; DOO, died of other causes; ND, not described; NED, (alive with) no evidence of disease.

## Discussion

Cyst excision is the standard of care for congenital choledochal cysts.^6^,^7^ Although sporadic cases of subsequent biliary malignancy developing in the remnant bile duct after cyst excision have been reported,^8^–^30^ there is a paucity of clinical evidence regarding the risk of subsequent biliary malignancy in patients undergoing cyst excision for congenital choledochal cysts. This prompted us to conduct the current study at a single institution. This is the first to demonstrate that the risk of subsequent biliary malignancy in patients undergoing cyst excision for congenital choledochal cysts seems to be relatively high in the long-term.

Coexisting carcinomas most commonly arise in choledochal cysts classified as types I and IV and are uncommon in types II, III, and V.^3^ Todani *et al*.^32^ reported that the risk of carcinoma remains, perhaps related to dysplasia and metaplasia of the epithelium. Therefore, complete cyst excision is essential to prevent subsequent biliary malignancy for congenital choledochal cysts with APBDJ. In type I and IV cysts, cyst excision involves complete excision of the bile duct from the confluence of the hepatic duct proximally up to the pancreaticobiliary junction distally.^18^,^30^,^33^ Biliary-enteric anastomosis at larger caliber duct is recommended for prevention of postoperative biliary strictures and subsequent biliary malignancy following cyst excision.^34^ It is also necessary for hepatobiliary surgeons to be careful not to damage the main pancreatic duct when the intrapancreatic portion of the dilated bile duct is excised.

The incidence of subsequent biliary malignancy after cyst excision was 4.3% in the current series. Some studies of subsequent biliary malignancy after cyst excision have reported incidence rates from 0.7% to 5.4%.^23^,^30^,^35^ Tocchi *et al*.^36^ reported that chronic inflammatory changes consequent to biliary-enteric anastomosis for benign biliary diseases should be closely monitored for the late development of biliary malignancies, suggesting that the risk of subsequent biliary malignancy may be associated with bilioenteric anastomosis itself. As the development of biliary cancer after cyst excision may depend on follow-up time, we applied the Kaplan–Meier method to estimate the risk of subsequent biliary malignancy following cyst excision. New results from our current study found that the cumulative incidences of subsequent biliary malignancy were 1.6% at 15 years, 3.9% at 20 years, and 11.3% at 25 years after cyst excision, suggesting that the risk of biliary malignancy in the remnant bile duct increases more than 15 years after cyst excision. Therefore, long-term follow-up is recommended for patients undergoing cyst excision for congenital choledochal cysts.

In the current series, the overall cumulative survival rates after treatment in four patients with biliary tract cancer were 50% at 2 years post-treatment and 25% at 3 years post-treatment, with a median survival time of 15 months (Fig. 2). A review of the literature revealed that there were no 4-year survivors among 32 patients with subsequent biliary malignancy following cyst excision. In 14 of these patients who received treatment for subsequent biliary malignancy, the overall cumulative survival rates after treatment were 32% at 2 years post-treatment and 16% at 3 years post-treatment, with a median survival time of 15 months (Fig. 3). Despite an aggressive treatment approach, subsequent biliary malignancy shows an unfavorable outcome. Patients undergoing resection for subsequent biliary malignancy are therefore clear candidates for adjuvant chemotherapy such as cisplatin plus gemcitabine.^37^

The main limitation of this study is the retrospective analysis of a small number of patients undergoing cyst excision for congenital choledochal cysts. The relatively small number of patients with subsequent biliary malignancy after cyst excision (*n* = 4) limits firm conclusions being drawn. To our knowledge, however, this is one of the largest series with the longest follow-up time dealing with subsequent biliary malignancy following cyst excision for congenital choledochal cysts; in addition, we evaluated both the cumulative incidence of subsequent biliary malignancy and the cumulative patient survival rates after treatment using the Kaplan–Meier method.

In conclusion, the risk of subsequent biliary malignancy in patients undergoing cyst excision for congenital choledochal cysts seems to be relatively high in the long-term. The risk of biliary malignancy in the remnant bile duct increases more than 15 years after cyst excision. Despite an aggressive treatment approach for this condition, subsequent biliary malignancy following cyst excision for congenital choledochal cysts shows an unfavorable outcome.

## References

[1] Flanigan PD (1975). Biliary cysts. Ann. Surg.

[2] Flanigan DP (1977). Biliary carcinoma associated with biliary cysts. Cancer.

[3] Todani T, Tabuchi K, Watanabe Y, Kobayashi T (1979). Carcinoma arising in the wall of congenital bile duct cysts. Cancer.

[4] Tsuchiya R, Harada N, Ito T, Furukawa M, Yoshihiro I (1977). Malignant tumors in choledochal cysts. Ann. Surg.

[5] Komi N, Tamura T, Miyoshi Y, Kunitomo K, Udaka H, Takehara H (1984). Nationwide survey of cases of choledochal cyst. Analysis of coexistent anomalies, complications and surgical treatment in 645 cases. Surg. Gastroenterol.

[6] Ishibashi T, Kasahara K, Yasuda Y, Nagai H, Makino S, Kanazawa K (1997). Malignant change in the biliary tract after excision of choledochal cyst. Br. J. Surg.

[7] Lipsett PA, Pitt HA, Colombani PM, Boitnott JK, Cameron JL (1994). Choledochal cyst disease. A changing pattern of presentation. Ann. Surg.

[8] Thistlethwaite JR, Horwitz A (1967). Choledochal cyst followed by carcinoma of the hepatic duct. South. Med. J.

[9] Gallagher PJ, Millis RR, Mitchinson MJ (1972). Congenital dilatation of the intrahepatic bile ducts with cholangiocarcinoma. J. Clin. Pathol.

[10] Chaudhuri PK, Chaudhuri B, Schuler JJ, Nyhus LM (1982). Carcinoma associated with congenital cystic dilation of bile ducts. Arch. Surg.

[11] Yoshikawa K, Yoshida K, Shirai Y (1986). A case of carcinoma arising in the intrapancreatic terminal choledochus 12 years after primary excision of a giant choledochal cyst. Am. J. Gastroenterol.

[12] Yamamoto J, Shimamura Y, Ohtani I (1996). Bile duct carcinoma arising from the anastomotic site of hepaticojejunostomy after the excision of congenital biliary dilatation: a case report. Surgery.

[13] Fujisaki S, Akiyama T, Miyake H (1999). A case of carcinoma associated with the remained intrapancreatic biliary tract 17 years after the primary excision of a choledochal cyst. Hepatogastroenterology.

[14] Goto N, Yasuda I, Uematsu T (2001). Intrahepatic cholangiocarcinoma arising 10 years after the excision of congenital extrahepatic biliary dilation. J. Gastroenterol.

[15] Koike M, Yasui K, Shimizu Y (2002). Carcinoma of the hepatic hilus developing 21 years after biliary diversion for choledochal cyst: a case report. Hepatogastroenterology.

[16] Ono S, Sakai K, Kimura O, Iwai N (2008). Development of bile duct cancer in a 26-year-old man after resection of infantile choledochal cyst. J. Pediatr. Surg.

[17] Shimamura K, Kurosaki I, Sato D (2009). Intrahepatic cholangiocarcinoma arising 34 years after excision of a type IV-A congenital choledochal cyst: report of a case. Surg. Today.

[18] Nagorney DM, McIlrath DC, Adson MA (1984). Choledochal cysts in adults: clinical management. Surgery.

[19] Rossi RL, Silverman ML, Braasch JW, Munson JL, ReMine SG (1987). Carcinomas arising in cystic conditions of the bile ducts. A clinical and pathologic study. Ann. Surg.

[20] Joseph VT (1990). Surgical techniques and long-term results in the treatment of choledochal cyst. J. Pediatr. Surg.

[21] Young WT, Thomas GV, Blethyn AJ, Lawrie BW (1992). Choledochal cyst and congenital anomalies of the pancreatico-biliary junction: the clinical findings, radiology and outcome in nine cases. Br. J. Radiol.

[22] Yamataka A, Ohshiro K, Okada Y (1997). Complications after cyst excision with hepaticoenterostomy for choledochal cysts and their surgical management in children versus adults. J. Pediatr. Surg.

[23] Kobayashi S, Asano T, Yamasaki M, Kenmochi T, Nakagohri T, Ochiai T (1999). Risk of bile duct carcinogenesis after excision of extrahepatic bile ducts in pancreaticobiliary maljunction. Surgery.

[24] Jan YY, Chen HM, Chen MF (2000). Malignancy in choledochal cysts. Hepatogastroenterology.

[25] Mabrut JY, Partensky C, Gouillat C (2007). Cystic involvement of the roof of the main biliary convergence in adult patients with congenital bile duct cysts: a difficult surgical challenge. Surgery.

[26] Palanivelu C, Rangarajan M, Parthasarathi R, Amar V, Senthilnathan P (2008). Laparoscopic management of choledochal cysts: technique and outcomes—a retrospective study of 35 patients from a tertiary center. J. Am. Coll. Surg.

[27] Kawarada Y, Das BC, Tabata M, Isaji S (2009). Surgical treatment of type IV choledochal cysts. J. Hepatobiliary Pancreat. Surg.

[28] Shah OJ, Shera AH, Zargar SA (2009). Choledochal cysts in children and adults with contrasting profiles: 11-year experience at a tertiary care center in Kashmir. World J. Surg.

[29] Lee SE, Jang JY, Lee YJ (2011). Choledochal cyst and associated malignant tumors in adults: a multicenter survey in South Korea. Arch. Surg.

[30] Takeshita N, Ota T, Yamamoto M (2011). Forty-year experience with flow-diversion surgery for patients with congenital choledochal cysts with pancreaticobiliary maljunction at a single institution. Ann. Surg.

[31] Todani T, Watanabe Y, Narusue M, Tabuchi K, Okajima K (1977). Congenital bile duct cysts: classification, operative procedures, and review of thirty-seven cases including cancer arising from choledochal cyst. Am. J. Surg.

[32] Todani T, Watanabe Y, Toki A (1987). Carcinoma related to choledochal cysts with internal drainage operations. Surg. Gynecol. Obstet.

[33] Ando H, Kaneko K, Ito T (1996). Complete excision of intra-pancreatic portion of choledochal cysts. J. Am. Coll. Surg.

[34] Kim JW, Moon SH, Park do H (2010). Course of choledochal cysts according to the type of treatment. Scand. J. Gastroenterol.

[35] Watanabe Y, Toki A, Todani T (1999). Bile duct cancer developed after cyst excision for choledochal cyst. J. Hepatobiliary Pancreat. Surg.

[36] Tocchi A, Mazzoni G, Liotta G, Lepre L, Cassini D, Miccini M (2001). Late development of bile duct cancer in patients who had biliary-enteric drainage for benign disease: a follow-up study of more than 1000 patients. Ann. Surg.

[37] Valle J, Wasan H, Palmer DH (2010). Cisplatin plus gemcitabine versus gemcitabine for biliary tract cancer. N. Engl. J. Med.

